# Retroesophageal right subclavian artery associated with a bicarotid trunk and an ectopic origin of vertebral arteries

**DOI:** 10.1007/s00276-021-02746-1

**Published:** 2021-04-15

**Authors:** Barbara Buffoli, Vincenzo Verzeletti, Lena Hirtler, Rita Rezzani, Luigi Fabrizio Rodella

**Affiliations:** 1 Division of Anatomy and Physiopathology, Department of Clinical and Experimental Sciences, University of Brescia, V.le Europa 11, 25123 Brescia, Italy; 2 Interdepartmental University Center of Research “Adaption and Regeneration of Tissues and Organs (ARTO)”, University of Brescia, Brescia, Italy; 3 Division of Anatomy, Center for Anatomy and Cell Biology, Medical University of Vienna, Vienna, Austria

**Keywords:** Anatomic variations, Aortic arch, Embryological anomalies, Retroesophageal right subclavian artery, Ectopic vertebral artery

## Abstract

A rare branching pattern of the aortic arch in a female cadaver is reported. An aberrant right subclavian artery originated from the distal part of the aortic arch and following a retroesophageal course was recognized. Next to it, from the left to the right, the left subclavian artery and a short bicarotid trunk originating the left and the right common carotid artery were recognized. An unusual origin of the vertebral arteries was also identified. The left vertebral artery originated directly from the aortic arch, whereas the right vertebral artery originated directly from the right common carotid artery. Retroesophageal right subclavian artery associated with a bicarotid trunk and ectopic origin of vertebral arteries represents an exceptional and noteworthy case.

## Introduction

The typical aortic arch (AA) branching pattern consists of three arteries: brachiocephalic trunk (BCT), left common carotid artery (LCCA) and left subclavian artery (LSA). The BCT is further divided into the right common carotid artery (RCCA) and the right subclavian artery (RSA). Several variations in the number and order of these branches have been described [1, 6, 8, 12]. One of the most common anomalies is the aberrant right subclavian artery (ARSA) or “arteria lusoria”, which has an incidence from 0.16% up to 4.4% in the general population with a female predominance [10], and a highest frequency in patients with congenital defects (up to 3%) and Down syndrome (up to 35%) [2, 10]. In this anomaly, the RSA arises independently from the descending aorta, instead of its normal origin from the BCT and it follows a retroesophageal course in 80–84%, a course between trachea and esophagus in 12.7–15%, and a pretracheal course in 4.2–5% [9, 10].

Among the main collateral branches of the RSA and LSA, there are the vertebral arteries (VAs), a couple of ascending vessels with a posterolateral course that enters the transverse foramen of the sixth cervical vertebra ascending in the neck and entering the cranium via the foramen magnum. Variations in the origin of the VAs are reported in the literature [8]. The most common VA variant is the left VA (LVA) arising directly from the AA (0.79–8%) [8, 11], whereas for the right VA (RVA) from the RCCA was reported with a low incidence (0.18%) [3].

There is some evidence about aberrant RVA and LVA origin coexisting with the ARSA; Lazaridis et al. [8] in their systematic literature review about the variability of VA, affirmed that, in presence of ARSA, the RVA originated frequently from the RCCA and the LVA from the AA. Gluncic et al. [3] reported an unusual origin of both VAs directly from the common trunk of VA and SA, on the left, and RCCA on the right. However, an ectopic origin of bilateral VA coexisting with ARSA and a bicarotid trunk (biCT), represents a very rare anatomical variant that was described mainly radiologically, during magnetic resonance angiography, or by schematic diagram [3, 5, 8, 11, 13, 15].

In this case report, we present a rare branching pattern of the aortic arch, with retroesophageal right subclavian artery associated with biCT, that should be considered by clinicians during their medical practice.

## Case presentation

An anatomic variation in the branching pattern of the AA was observed during a student’s routine dissection course of a Caucasian female cadaver at the Anatomical Training Center of the University of Brescia. The specimen was donated by the voluntary body donations program of the Center for Anatomy and Cell Biology of the Medical University of Vienna. The donor provided written informed consent prior to death for the body use in medical education and research.

**Fig. 1:**
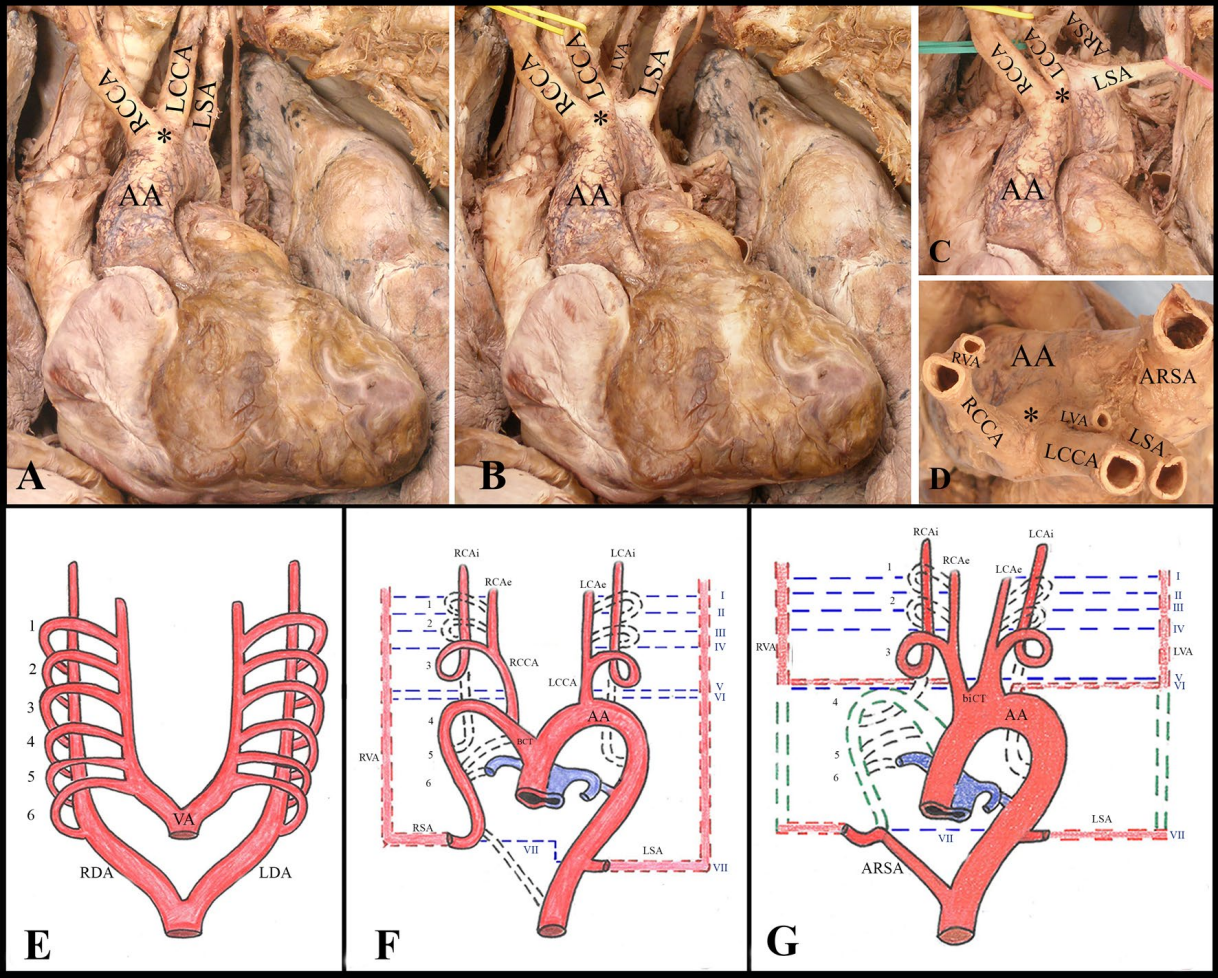
AA branching pattern: **a** biCT that gives the RCCA and LCCA, and the LSA are visible; **b** LVA is observable, moving the LCCA; **c** ARSA is evident, moving the LCCA and the LVA; **d** upper view of the branching pattern: RVA arising from the RCCA and LVA from the AA are visible. Schematic representation: **e** six pairs of aortic arches during early embryonic development; **f** normal embryologic development of the aorta and brachiocephalic vessels; **g** hypothesized abnormal embryological development of this anomalous branching pattern. Abbreviations: *AA* aortic arch, *ARSA* aberrant right subclavian artery, Asterisk indicates *biCT* bicarotid trunk, *LDA/RDA* left/right dorsal aorta, *LCAe/RCAe* left/right external carotid artery, *LCAi/RCAi* left/right internal carotid artery, *LCCA/RCCA* left/right common carotid artery, *LSA/RSA* left/right subclavian artery, *LVA/RVA* left/right vertebral artery, *VA* ventral aorta, (1–6) aortic arches; (1–7) intersegmental arteries. Black dashed lines: regression of the aortic arches. Blue dashed line: regression of the intersegmental arteries. Green dashed line: normal embryological pattern (color figure online)

**Fig. 2:**
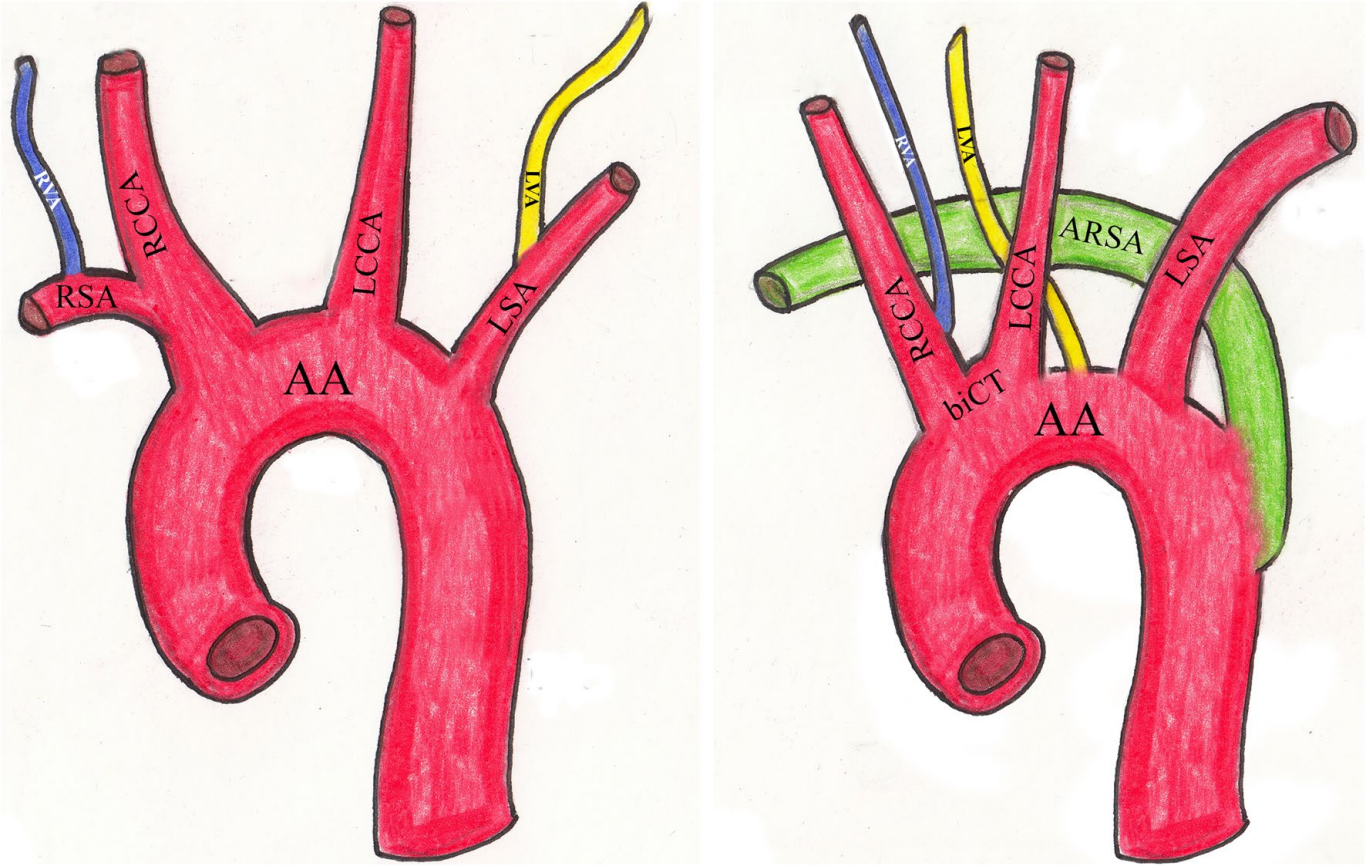
Schematic drawing: **a** normal AA branching pattern; **b** anomalous AA branching pattern, showing anatomic variation. Abbreviations: *AA* aortic arch, *ARSA* aberrant right subclavian artery, *biCT* bicarotid trunk, *LCCA/RCCA* left/right common carotid artery, *LSA* left subclavian artery, *LVA/RVA* left/right vertebral artery

All the dissection procedures were led to taking care of the surrounding soft tissues, vessels, nerves, and muscles. AA was exposed after removal of the anterior thoracic wall, fat tissue, and the pericardium covering the ascending aorta and the great vessels. An unusual variation in the branching pattern of the AA was noted (Fig. 1). BCT was not found and a short biCT, such as a bulge formed by the union of enlarged origins of RCCA (6.35 mm) and LCCA (6.79 mm) neighboring with each other, was identified from the AA. Next to it, from the right to the left, the LSA (6.8 mm) and, more distally, the ARSA (9.2 mm) with a retroesophageal course. In addition, an ectopic origin of bilateral VAs was found. The RVA (3.2 mm) arose from the RCCA and entered the transverse foramen of the fourth cervical vertebra, whereas the LVA (3.0 mm) developed directly from the AA, between the LCCA and the LSA, and entered the transverse foramen of the fifth cervical vertebra (Fig. 2). Other variations were not found.

## Discussion

Detailed knowledge of the embryological development of the AA and its branching pattern is a primary requirement for a full awareness of these vascular variations. The AA and its branches develop during the 1st week of the intrauterine life and take their definitive shape in the 8th week. Six embryonic pairs of branchial arch arteries, arising from the aortic sac, are initially modeled and developed; most of all regress, and the residual branches form the AA and the great vessels. Just in this period, anatomical variants may ensue [8].

In this case, a rare anomalous branching pattern of the aortic arch associate with ARSA was found, suggesting additional combinations of embryological anomalies in the development of the AA. In particular, we observed retroesophageal ARSA, that represented the distal branch arising from the AA, and next to it, from the left to the right, the LSA and a short biCT, such as a bulge formed by the union of both the enlarged origins of the RCCA and LCCA.

Retroesophageal ARSA is one of the most common anomalies in the AA, as it has been recently reported by Natsis et al. who reported a prevalence of 8% [11]. From an embryological point of view, the proximal part of RSA derives from the right fourth arch, while the distal part derives from the right dorsal aorta and the right seventh intersegmental artery; differently, LSA derived entirely from the left seventh intersegmental artery. In event of the right fourth arch and the right dorsal aorta (proximal part) involution, the RSA forms from the right seventh intersegmental artery and from the distal part of the right dorsal aorta; in this case, the RSA becomes the “arteria lusoria” or ARSA [10]. About biCT, it represents another frequent branching pattern variation that coexists with ARSA with an incidence of 4–20.6% [11, 13]. From an embryological point of view, both carotid arteries arise from the third arch with a common trunk, and this common carotid origin variant could be just explained with the persistence of this stage.

In addition, from the right CCA and next to the biCT, we found the origins of the VAs: the RVA arose from the RCCA, whereas the LVA arose directly form the AA, between LSA and biCT. Ectopic origin of the VAs from the AA and RCCA have been reported with a very low incidence, even if their coexistence with ARSA is more frequent [2, 9, 11, 13]. About their embryological development, it is known that VAs appear late in the embryonic evolution (7–12 mm stage), from transverse anastomoses between the cervical intersegmental arteries [8]. Typically, the first part of these arteries originates from the distal end of the seventh intersegmental artery; but, if the third–sixth intersegmental artery persists an abnormal origin of the VA from the AA or from the CCA occurs [4, 7, 8].

The outcomes of this rare embryological scenario led to the described exceptional case, in which vascular variations of the main branches of the AA and an ectopic origin of both the VAs coexist. This was previously observed only in very few works [8, 9, 11, 14, 15], which testify the rareness of this work in the extensive panorama of the anatomical variations of this area.

About the clinical impact of these anatomic variations, the clinical syndrome of the ARSA is often associated with dysphagia, as termed “dysphagia lusoria” by Bayford in 1787. Reports have also described spontaneous rupture, dissection, increase risk of thrombosis, intracranial aneurysm formation, that may be linked to the turbulent flow in an aberrant origin. Other symptoms are stridor, retrosternal pain, cough, feeding difficulties accompanied with weight loss, recurrent pulmonary infections, stomachache, back pain, and numbness of the right upper limb [10]. Moreover, biCT is the commonest cause of tracheobronchial compression and the coexistence with retroesophageal ARSA could limit tracheal-esophageal mobility [10, 11]. Finally, about aberrant VAs, they have been often found in asymptomatic cases, and their symptoms usually appear when the atypical origin coexists with an aneurysm formation, aortic dissection, coarctation, and congenital heart diseases [4, 8]; however, their presence could lead to misdiagnosis and consequent endovascular and surgical complications.

We believe this noteworthy case represents a rare branching pattern of the AA found in an anatomical specimen, according to Lazaridis et al. [8] who reported, in a systematic classification of the VA variable origin, a very low incidence (0.38%) of aortic origin of LVA coexisting with biCT and ARSA. Therefore, this work represents a precious contribution in the whole comprehension of the anatomical branching variants of the AA including ARSA, biCT, and ectopic origin of the VAs. The knowledge of this rare anatomic pattern should be considered by radiologist, interventional cardiologists, and thoracic surgeons during their daily surgical, angiographic and clinical medical practice.
